# Navigating Support for Informal Caregivers of Older Migrants with Dementia in The Netherlands: Insights from Patient Journey Mapping

**DOI:** 10.3390/geriatrics11040105

**Published:** 2026-08-14

**Authors:** Margaret von Faber, Suzan van der Pas

**Affiliations:** 1 Faculty of Social Work & Applied Psychology, University of Applied Sciences Leiden, Zernikedreef 11, 2333 CK Leiden, The Netherlands; faber.von.m@hsleiden.nl; 2 Department of Public Health and Primary Care, Leiden University Medical Center/Health Campus, Spui 5, 2511 BL The Hague, The Netherlands

**Keywords:** patient journey mapping, dementia, informal care, older migrants

## Abstract

**Background/Objectives**: The aim of the study is to determine factors that affect informal caregivers’ ability to care for older migrants with dementia, with a broad aim of providing a framework for improving regional formal support. We used patient journey mapping as a method to identify experiences of informal caregivers. **Methods**: The study took place in three phases. In the first phase, the content of the patient journey was outlined, and semi-structured interviews were conducted with 14 informal caregivers of people with dementia and a migrant background. In the second phase, results of the interviews were discussed in workshops with professionals. In the third phase, professionals worked together on a plan for improving care and support. **Results**: Besides positive experiences with care and support, informal caregivers expressed the need for knowledge and training, timely information as well as practical culturally sensitive care and support. Professionals expressed a need for more knowledge of culturally sensitive issues and conversation techniques. They also indicated the need for more integrated care and the ability to offer practical solutions for informal caregivers. As a final result, a multidisciplinary programme on improving knowledge, information for clients, informal caregivers and professionals, use of key figures, culturally sensitive support, a multidisciplinary care path and education for future professionals was launched for the region. **Conclusions**: A patient journey is a valuable method to pinpoint problems and needs of informal caregivers as well as professionals. It provides a foundation for multidisciplinary communication and collaboration to improve care and support.

## 1. Introduction

In general, one in five people will suffer from dementia. However, among older people with a migrant background in The Netherlands, the percentage of people suffering from mild cognitive impairment and dementia is higher than in people with a Dutch background. In 2016, Parlevliet et al. found that mild cognitive impairment was two to three times more prevalent in Turkish, Moroccan and Surinamese–Hindustani populations compared to the native Dutch population, while dementia was three to four times higher in these populations [1]. In The Netherlands, the percentage of older people with a migrant background is increasing rapidly. By 2060, they are expected to constitute 21% of the population, compared with 11% in 2015 [2]. This means that the prevalence of older migrants with dementia will also increase [3].

Older people with a migrant background are a diverse group. They come from different countries of origin and have varying cultural backgrounds, religions, and a differing migration history. In The Netherlands, we can distinguish between several groups: a large number of older migrants come from former colonies of The Netherlands (Suriname, Indonesia, the Dutch Caribbean); another group comes from neighbouring countries like Germany; another group are the former guest workers from countries like Spain, Marocco and Turkey; and a specific and diverse group are the people with a refugee background from countries such as Iraq, the former Soviet Union, Iran, Afghanistan and Syria [4,5]. It is predicted that the population with a migrant background will become even more diverse in the future, because of changing patterns of immigration [6]. Compared to older people with dementia in the general population, older people with dementia and a migrant background are underrepresented in regional census data and national statistics.

In the disease trajectory of dementia, older people with a migrant background are often underdiagnosed and several factors, such as stigma, language or structural factors may hinder timely diagnosis [7,8,9]. In screening, obtaining access to culturally sensitive screening and diagnostic tools may be a challenge [9,10]. Informal caregivers may experience an increased burden due to a range of factors, including barriers to accessing and using culturally sensitive care [11], perceptions of “good care” and (gendered) norms of reciprocity, challenges regarding sharing the caregiving burden with family members [12], a combination of cultural, religious or social norms or social class, and the intensity of care. In a survey of informal caregivers who care for their parents, caregivers with a non-Western migration background provided approximately 7.5 h a week of care for their parents, compared to 5.1 h a week for informal caregivers of parents without a migration background [13]. Migrants tend to receive less end-of-life care in hospices or at home [14]. Compared to older people with dementia in the general population, ethnic minority groups use healthcare services less frequently and prefer alternative care arrangements. In particular, migrants with an Asian, Moroccan or Turkish background tend to choose a Personal Budget Grant (PGB) [4]. A PGB is a Dutch-based grant which enables individuals to make their own choices in caregiving and support, for example to hire a care professional that speaks the language of the person with dementia.

During the process of dementia, from the early signs of cognitive decline to the end of life, many different professionals may be involved in providing care and support. Patient journey mapping is a relatively novel research method that enables the description of patients’ or informal caregivers’ experiences across different phases of the disease trajectory and their interactions with professionals.

Journey mapping, also known as “process mapping”, “experience maps” or “customer experience maps”, is a research method developed from marketing professionals with the aim to improve services [15]. A patient journey can be used to inform the redesign and improvement of health service delivery; to develop a deeper understanding of a person’s journey through the health system(s); to identify gaps in care and unmet needs; to evaluate continuity of care across health services and regions; to understand the comprehensiveness of care; to understand how people are navigating through health systems; or to compare patient experiences with practice guidelines and standards of care [16]. There are different forms and methods of patient journey mapping [17,18,19]. Although a patient journey map can be used for improving specific parts of healthcare provision or the whole journey, general characteristics are the visualisation of the journey, the user perspective as an important point of reference and the aim of improving healthcare services [20].

In this paper, we describe the patient journey from the perspective of informal caregivers for individuals with dementia and a migrant background. In the Dutch language we used the word “client” instead of “patient” because it is used in Dutch welfare provisions and support (which includes social care and community care). We define the patient journey as “the steps an informal caregiver and a client take as they progress through different stages of a disease, often capturing diagnosis, management and interactions with health professionals” [21,22].

In this patient journey, the University of Applied Science Leiden worked together with Transmuralis, a coordinating organisation for healthcare and welfare in the Northern part of the South Holland region, which includes 13 municipalities. Transmuralis is an umbrella organisation comprising healthcare and social care providers, general practitioners (GPs), a regional expert group on dementia and the regional healthcare office [zorgkantoor].

Our goal was to identify positive experiences as well as unmet needs during the disease process of dementia from the perspectives of people with a migrant background and their informal caregivers. A second goal was to improve regional formal care and support for these informal caregivers in our region, as there are currently no care or welfare programmes tailored for individuals with dementia from migrant or ethnic minority back-grounds, as opposed to other regions in The Netherlands. To our knowledge, a patient journey method for this growing group of caregivers in The Netherlands has not yet been conducted.

## 2. Materials and Methods

### 2.1. Study Design

The study took place in three phases:

(a) Semi-structured interviews with informal caregivers.
(b) Reflection with professionals and generating new insights in workshops.
(c) Construction of a plan for improvement.

In the first phase, a multidisciplinary group of six professionals and volunteers with expertise in dementia care and welfare from different organisations in the region were involved in the outline of the journey, the recruitment of participants, design of the topic list for the interviews, information for professionals and participants, and the informed consent form for participants. Two members of this team had a migrant background themselves. Both authors MF (PhD) and SP (PhD) worked as professor [SP] and senior researcher [MF] at the Faculty of Social Work & Applied Psychology at the University of Applied Sciences Leiden. MF has professional experience in dementia care and has no migrant background. The researchers worked closely with the team members and the coordinator of the programme for dementia care of Transmuralis.

The content of the journey was outlined in six stages, in line with perspectives of people with dementia [23]: 1. the first signs; 2. undertaking action (receiving a diagnosis); 3. first contact with professional support; 4. living at home with dementia; 5. complex care at home/in a nursing home; 6. end-of-life care and aftercare for the family.

The first author conducted semi-structured interviews with informal caregivers of individuals with dementia and a migrant background. The topic list was pilot-tested and included general characteristics of the participants and cultural background of the person with dementia (the topic list is available in the Supplementary Material). The participants were stimulated to elaborate on the present stage of caregiving, presence of other informal caregivers and their roles in caregiving [24]. Then, in retrospect, the first signs of dementia, the way in which the person involved and the social network responded was elaborated on towards the current stage in the journey. Next, the respondents were asked about their expectations, concerns and ideas for the future. If the person with dementia had passed away, the topic of expectations for the future development of care was skipped. The interview ended by asking for recommendations for other informal carers and professionals. Feelings and emotions during the journey were listed. Field notes were made after the interviews. Data saturation was discussed with team members and the coordinator of Transmuralis.

In the second phase, results of the interviews were discussed with professionals in healthcare and welfare during three workshops. In the first two workshops, we created two personas to discuss available support, limitations and suggestions for improvement. The personas were based on the results of different interviews. One persona was a man with no family in The Netherlands who took care of two parents, with a PGB. The other persona was a young woman who took care of her father together with her family and who experienced internal conflicts within the family. Dementia case managers, a geriatric physician, a policy maker, nurses, informal care coaches, activity coordinators in daycare for people with dementia and a manager in a welfare organisation discussed their experiences in small groups and wrote comments, limitations and suggestions on sticky notes for each stage of the journey, outlined for each persona on flipchart papers. The third workshop brought together managers and policy officers from municipalities, education, healthcare, and welfare sectors, as well as representatives from the regional care office. They were invited to set up short-term and long-term goals for practical improvements on major themes.

In the third phase, regional plans were made to improve care and support. The regional expert advisory group on dementia from Transmuralis discussed the available options in the regional context. By including the actions in policy documents, the implementation of the improvement plan will be ensured. We organised a meeting to disseminate the results for professionals and study participants and students to inform them about the regional improvement plan.

### 2.2. Sampling and Participants

Although we initially aimed to include individuals with dementia, as we did in our earlier research among individuals with a Dutch background [25,26], this proved very difficult. Informal caregivers did not give their consent for the individuals with dementia they cared for to be interviewed because they stated it would be too tiring or too difficult for the person involved. Therefore, we chose to recruit a greater number of informal caregivers instead by convenience sampling.

Participants were notified about the research through leaflets in community centres and shops. The leaflet included a photograph of the researcher and explicitly stated that all data collected would be handled in strict confidence. Additionally, participants were invited by key informants and professionals such as case managers, and provided with information about the study, an invitation letter and a summary of the study.

Informal caregivers were eligible as participants when they cared for a person aged 65 and over who was not born in The Netherlands. As dementia is not a familiar term in some cultural contexts, and people with a migrant background are often underdiagnosed, we described it as caring for someone with signs of memory loss, changed behaviour or dementia. A formal diagnosis was not required; however, during the first telephone contact, the researcher asked about other potential causes of changed behaviour. The informal caregivers could be involved in different stages of the journey, up to a year after the death of the person with dementia. Care and services had to be received in the region in order for the research team to assess them.

When they consented to participate, their contact details were shared with the researcher [MF] and they received an invitation for an interview via a telephone call from the researcher. There was no relationship between researcher and participants prior to the study.

In total, 20 informal caregivers gave their initial consent for an interview. One informal caregiver who took the initiative to contact the researcher was excluded because her father suffered from a cerebrovascular accident and had no signs of dementia. Five informal caregivers decided not to participate in an interview for different reasons, which included: (i) a language concern from a person with a Nepalese background, (ii) a mother that did not want her son to participate, (iii) two caregivers mentioned recent changes in caregiving and feeling overburdened, and (iv) concern about emotions after the recent loss of a father. Data were collected in the period of October 2023 until July 2024.

Participants were informed about the explorative aim of the study and signed the informed consent form prior to the interview. All participants were interviewed once. The interviews lasted about 1.5 h each and were conducted in the participant’s home or a place of their choice. In one interview at a participant’s home, a father with dementia was sleeping on the couch during the whole interview. All interviews were audio-recorded, transcribed, and securely stored in the institutional cloud of the principal investigator. Transcripts of the interviews were not returned to participants for comments or correction, as they preferred to receive the final results of the study. The report summarising the study findings [27] was shared with the participants and they were invited to a conference presenting the study findings and the regional improvement plan.

### 2.3. Data Analysis

Analysis of the data was based on an inductive approach according to the principles of grounded theory [28]. Thematic content analysis of the interviews with informal carers was performed with Atlas Ti 23 (ATLAS.ti Scientific Software Development GmbH, Berlin, Germany). A coding scheme was developed and tested in an iterative fashion. The codes were grouped into themes linked to the six stages of the journey, such as the first signs of dementia or experiences in getting a diagnosis. The positive and negative experiences in the stages of the journey were coded, as well as needs or recommendations for improvements and suggestions for other informal caregivers. Data were coded by the first author and discussed with the coordinator of Transmuralis.

Data from the two workshops with professionals were analysed by the first author and the coordinator of Transmuralis by structuring the comments on sticky notes on the flipcharts for each persona into: professional contacts, hurdles, needs and suggestions for improvement in each stage of the patient journey. Finally, the needs for improvement were labelled in six themes: improving competence for professionals; improving information for patients, caregivers and professionals; culturally sensitive care provisions; integrated care; use of key persons with different cultural backgrounds, and education for future professionals.

## 3. Results

### 3.1. Sociodemographic Characteristics of Informal Caregivers

We conducted interviews with 14 informal caregivers. The informal caregivers included 11 women and three men, ranging from age 33 to 84 (see Table 1).

Besides providing informal care, eight participants combined their caring with employment, and six participants had their own family with [young] children living at home. Five participants lived with the individual[s] they cared for in the same house. Five participants cared for two parents at the same time. Four participants arranged elements of care with a PGB, which allowed them to hire specific care providers or to use this budget for themselves in providing 24 h care.

### 3.2. The Visualisation of the Patient Journey

**Figure 1:**
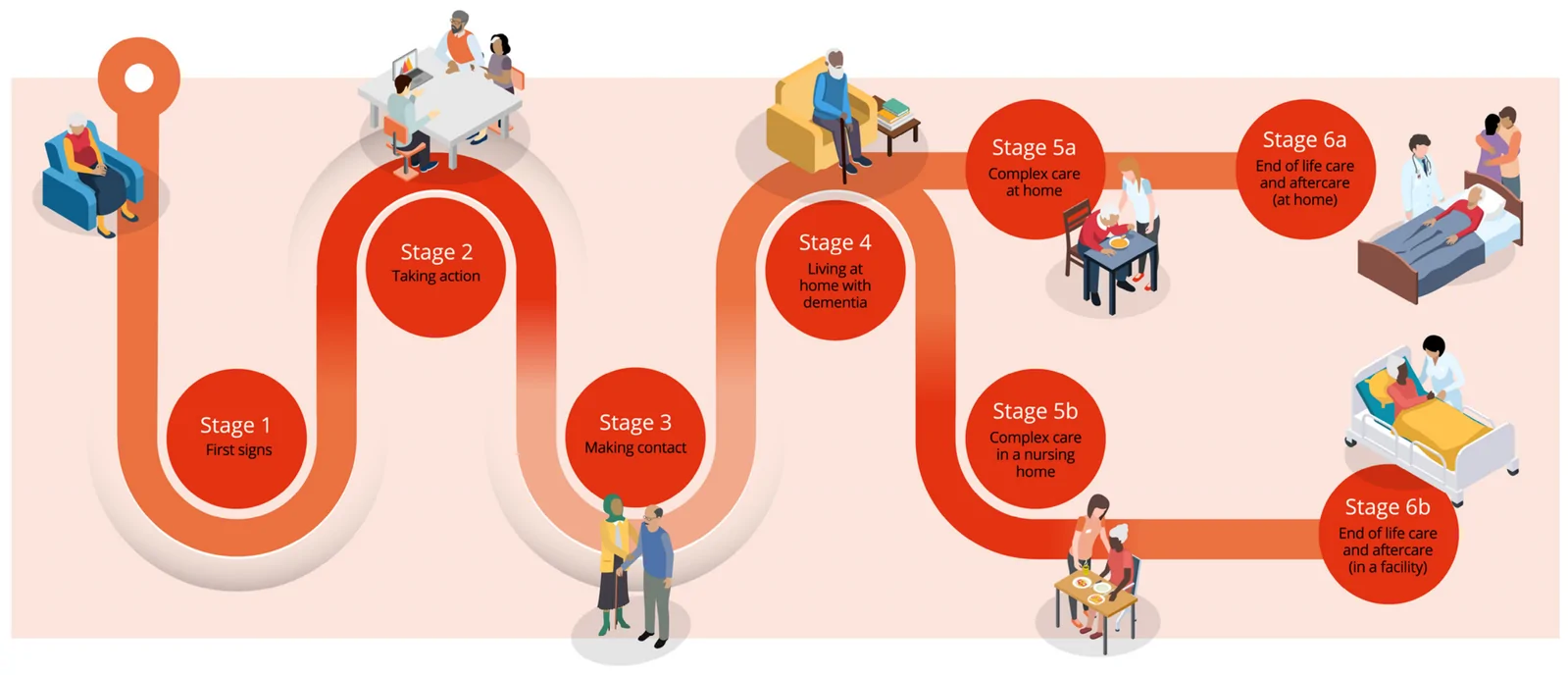
The patient journey of informal care for individuals with dementia and a migrant background. Stage 1: First signs. During the first stage, changes in behaviour or daily life become apparent for the social network of a person, the individual with dementia or a psychiatrist. Stage 2: Taking action. In the second stage, the social network of a person often undertook action to consult a GP, a geriatric nurse or geriatric specialist, or mental healthcare for a diagnosis. Stage 3: Making contact. In the third stage, informal caregivers came into contact with welfare or healthcare professionals, for example the case manager or informal care coaches. Stage 4: Living at home with dementia. Stage four is characterised by the patient living at home with available support. A broad range of professionals can be involved, depending upon the circumstances of the individual with dementia and their informal caregivers. This support can vary from general information provided by municipal services regarding home adaptations to case management, caregiver training programmes, home care services, specialised professional support (such as physiotherapists and occupational therapists), and contact with health insurance providers. Stage 5: Complex care. In stage five, there is a need for extensive care because the mental and physical condition of the person with dementia has deteriorated. Often day and night care is needed for the person with dementia. In this stage, family members must decide whether or not extensive care is provided at home (5a) or their family member is admitted to a nursing home (5b). Stage 6: End-of-life care and aftercare. Stage six is characterised by terminal care, care for the deceased person in the home situation (6a) or in an institution (6b). The funeral is in The Netherlands or in the country of origin. Aftercare can be provided for the next of kin and is part of institutional care.

Based on the interviews with the informal caregivers, we created a visualisation of the journey (Figure 1).

### 3.3. Perspectives from Informal Caregivers and Professionals

#### 3.3.1. Positive Experiences from Informal Caregivers

Participants discussed aspects of professional support that they experienced as helpful and positive. With regard to stage 2 (taking action), two respondents mentioned the effort of their GP to find a physician that spoke Moroccan and could test in a way their parent could understand. Another participant mentioned the efforts of the GP in stage 3 (making contact) in finding a home-care service with staff from the same cultural background. With regard to stage 4 (living at home with dementia), several respondents mentioned positive remarks about the support of a case manager for people with dementia in explaining and providing information about the process, practical solutions and helping with procedures:

*“I got the feeling that I could always contact her, in case of questions”*

[R05]

Advice about maintaining mobility from a physiotherapist who visited the person, who had both dementia and rheumatism, at home, was also perceived as very positive. Another participant stated that receiving the message that a personal budget (PGB) was granted made her shed tears of happiness.

*“To go along with them [parents] to all the appointments, I took time off and used up all my vacation days. There was no other option.”*

[R08]

The PGB enabled her to find a professional for home care and support that spoke Russian, the language of her parents and her father with dementia.

Several participants mentioned the support of specific organisations for home care or mental healthcare that were not part of the regular and widely recognised care in the region.

“*The Sanctuary Foundation [Stichting Sanctuary]. That is …there are a psychologist, a psychiatrist and a social worker all with a migrant background.”*

[R01]

*“We don’t know all that”*

[MF]

*“Yes. That’s the problem too, isn’t it? Because there is a lot, but one doesn’t know about the other and I think we can achieve a lot if we work together.”*

[R01]

Participants had received this information through their social network, a GP, or had found it on the internet.

#### 3.3.2. Needs and Bottlenecks from the Perspectives of Informal Caregivers

Besides positive experiences, participants also mentioned needs and lack of support. In the stage of the first signs of dementia, most participants mentioned that they had little knowledge about dementia and difficulty finding appropriate information. Although the younger generation, raised in The Netherlands, perceived dementia as a disease, the older generation was not always familiar with dementia and did not always understand the clinical picture.

*“My mother still… I can’t explain it to her. I do try to explain what dementia is. She always says: he is crazy!”*

[R12]

*“They don’t use the word ‘dementia’ in Surinam. ’I’m getting old and forgetful.’”*

[R13]

However, there was considerable diversity in how families discuss dementia, depending on the culture and country of origin.

*“I suppose that the Dutch can talk about dementia more easily and openly than people with an Eastern background*.”

[R07]

In the stage of receiving the diagnosis, informal caregivers reported the need for professionals to show patience and respectful treatment in the use of diagnostic tests, instead of incorrectly assuming a stereotypical image of guest workers:

*“[…] because many Moroccan men have been guest workers, they may have had no prior training, so it was already assumed that they have a low educational level. But that says nothing about their IQ. So it was also the way he was treated, she [professional] was very much biased in a negative sense.”*

[R12]

Some informal caregivers mentioned comorbidity of dementia and Post-Traumatic Stress Disorder (PTSD) due to traumatic events:

“*[…] my father was therefore referred to mental health services [GGZ]. Partly to investigate how things stand with his cognitive functions and, at the same time, in connection with severe trauma from the past, because the trauma had never really been processed. […] considering the events of a year ago … Since then, even our country, the country where we were born… no longer exists. That caused the trauma to come sharply back to the forefront.”*

[R08]

*“And my father already had a therapist at the mental health services [GGZ] for his anxiety disorder. So I raised the alarm there, I also noticed that his panic was getting worse. He started experiencing more panic and anxiety symptoms. That therapist immediately thought of dementia.”*

[R14]

In stage 3, after the diagnosis was given, all informal caregivers reported the need for information. They needed information on the process of dementia, what to expect, but also available professional support and their own role or responsibilities. They also reported the need for explanation of all the different regulations and arrangements with their acronyms, the different professionals involved and the advantages and disadvantages of the regulations.

*“It was a long search: what is possible? Who could do what? What was I supposed to do as a caregiver?”*

[R10]

In stage 4 (living at home with dementia), there was a need for practical support, professional homecare with an open mind for cultural demands, such as taking off the shoes when entering a home. Also, there was a need for training, culturally sensitive daycare facilities, in a Mosque or in an institution, and support or care during the night. Often family members took care of a parent at night, alongside their jobs during daytime. Respite care was difficult to find:

“*That’s what I missed. My search for respite care led to nothing. Because there was no… the language barrier.”*

[R06]

The role of the mosque was mentioned several times:

*“In the mosque it [dementia] is not a topic talked about that much. Unfortunately. At this moment we [respondent with professional of welfare organisation] are discussing: what can we do for this generation? Can we break the taboo around it? It’s not wrong to talk about it.”*

[R02]

Because informal care is sometimes a family responsibility, there was a need for a family conversation with professionals instead of a single contact representing the family in discussions with professionals. Admission to a nursing home is not usual because of the language barriers:

*“There should be more wards for migrants in nursing homes. If I leave my father there alone, he’s going to die. He can’t talk to anyone all day.”*

[R10]

Especially when care becomes increasingly intensive and complex, in stage 5 and 6, informal caregivers wanted consultation and support from professionals:

*“When homecare workers notice that strange things are happening, they should take the initiative to take action instead of thinking that the family will solve it by themselves.”*

[R02]

*“We should discuss it together. How should the care be provided [in the future]? How do we as family fit in? But she only fills in the pieces for her organization: “We can offer this package.””*

[R13]

Although participants indicated general cultural guidelines or wishes of the individual with dementia, sharing care with professional caregivers in terminal care appeared to be a topic on which most participants had not yet formed an opinion. Only two informal caregivers had experienced the terminal care stage, so it was only briefly discussed. For one respondent, it became an emotional topic because family members had suddenly moved the person with dementia to Marocco without telling the other caregivers. She was angry and still grieved because she did not have the chance to bid a last farewell.

During the patient journey several emotions come to the fore: acceptance of the diagnosis, a long search for appropriate support and uncertainty, feelings of filial obligation but also feelings of frustration and feeling overburdened especially in the stage of complex care at home:

*“It all became too much. I said to my husband: “I’ll stop. I am not obliged to do that [informal care]. I am obligated in Islam to care for my mother and father. As a daughter-in-law I am not obliged to do that!” He understood it well, but he couldn’t take it anymore himself. Then I thought: I will also have a husband with a burnout at home. So what to do? Then I picked up care again. Reluctantly.”*

[R02]

When asked for advice to give to other informal caregivers the following themes were mentioned: breaking the taboo of asking for help, searching for support and available care, contacting the GP, not postponing important decisions, and for caregivers to take good care of themselves. Two informal caregivers mentioned training courses and meeting other informal caregivers. The youngest informal caregiver mentioned social media, in which an influencer shares her experiences in caring for her father with dementia and a migrant background and knowledge about the illness is shared.

Table 2 lists the needs and bottlenecks from the perspective of informal caregivers.

#### 3.3.3. Needs and Bottlenecks from the Perspectives of Professionals

In the three workshops, a total of 30 professionals from 20 different organisations relating to education, finance, municipality, health and welfare on a local or regional level participated.

In the first two workshops, professionals discussed, using personas, the challenges faced by informal caregivers across the different stages of care, as well as their own experiences and recommendations in these domains. The professionals stated that they recognised many of the bottlenecks experienced by the informal caregivers. Although professionals endeavoured to provide relevant and practical support for the person with dementia and the informal caregiver, they also stated that they sometimes felt unable to act due to the fear that they were not well equipped for the task. They sometimes felt unable to build a relationship of trust with families. They expressed the need for information about cultural backgrounds in relation to perspectives on dementia; dealing with dementia and traumatic experiences for example for migrants with a life history as a refugee; and cultural perspectives on palliative care and conversation techniques with a family. Professionals stated that there is often a lack of knowledge among nurses in nursing homes of trauma processing (PTSD) in combination with dementia. Professionals all expressed the need for more culturally sensitive facilities and solutions in order to be able to offer person-centred care. Table 3 lists the needs and bottlenecks from professionals’ points of view in the stages of the patient journey.

Professionals mentioned that a social map of welfare and care providers would help in the finetuning between different professionals such as the GP, the case manager and ambulatory care provider. They came up with several solutions such as improving the use of assistive home technology [domotica] in the home situation and increasing familiarity with training for informal caregivers.

### 3.4. Solutions and Plan for Improvement

In the third workshop with professionals, several themes were found for improvement. These themes were discussed with professionals on a strategic level, in order to make a plan that could benefit both informal caregivers and professionals. Agreement was reached in the regional dementia network on six themes with several actions:

The first theme is “Information for clients and professionals”. A working group will draw up an inventory of relevant national and local information; the information will be linked with healthcare insurance and specific healthcare providers (like mental healthcare institutions); options for digital information or information on paper will be investigated and a plan for implementation will be finalised. The existing regional website for information on dementia will get an option for translation into different languages.

The second theme is the use of “Key figures”. Key figures are individuals who have both a migration background or a history as a refugee and are familiar with the Dutch welfare and healthcare system. A working group will explore the available user experience experts and create conditions for the regional use of these experts.

The third theme is “Increasing knowledge among current professionals”. In 2025, the results of this research were shared and courses have been scheduled for professionals on: conversation techniques with a family; processing of traumatic life events in combination with dementia; and cultural perspectives of dementia and cultural aspects of terminal and palliative care.

The fourth theme is “Practical culturally sensitive support”. Cooperation partners from different organisations will develop a plan for increasing the use of available assistive home technology [domotica] by informal caregivers; and setting up daycare for women with dementia and a migrant background; raising awareness about training for informal caregivers on caregiving and increasing the support of caregiver support agents [mantelzorg-ondersteuners].

In the fifth theme, a working group will develop a multidisciplinary care path [zorgpad]. The care path must provide knowledge about and collaboration between GPs, other healthcare professionals, and professionals in the social domain and municipalities in the region. Contact people of mosques can be added, as well as key figures in welfare and care with a migrant background. There is an example of a care path for migrants with dementia in another region and the working group can use this as a format. The care path will be linked with two other care paths already used by GPs: the care path for frail elderly people and informal caregivers.

The sixth theme is “Education for future professionals”. Because of increasing population diversity, cultural competence in vocational education is seen as imperative by current professionals. The University of Applied Sciences can be responsible for improving the education for future professionals in social work, nursing and applied psychology.

The regional expert advisory group on dementia from Transmuralis discussed the available options in the context of other priorities and shortages in the workforce. Therefore, a regional plan instead of a local plan for different organisations was launched. The actions were included in policy documents for 2026–2027. Some actions started immediately, like the translation of the regional website. Transmuralis will take the final responsibility for the execution of the improvement plan.

## 4. Discussion

This study focused on informal caregivers for people with dementia and a migrant background. Although managing dementia is demanding and may be difficult for families independent of their cultural background, the context of available resources and problems concerning language and cultural or religious background can provide an extra challenge for appropriate care and support. There can be various obstacles in the use of welfare and healthcare from the part of informal caregivers [11].

Patient journey mapping can be applied to a range of objectives [16]. In this study, it was used to identify gaps and unmet needs in formal care and support for people with dementia from a migrant background and their informal caregivers by exploring caregivers’ experiences across the different stages of dementia.

As patient journey mapping encompasses diverse approaches [17,18,19], there is no standardised framework for its visualisation, terminology, or reporting. Transparent reporting of the methodology is therefore essential [16]. We structured our approach into three phases: semi-structured interviews with informal caregivers, reflective workshops with professionals, and the development of an improvement plan. This enabled us to examine positive experiences, challenges, needs, and wishes from the perspectives of informal caregivers while also considering the experiences and views of professionals.

The journey-mapping method as conducted in our study can be used for a multidisciplinary approach to improve support and care. In the context of the Northern part of the South Holland region, this patient journey research was a collaboration between the University of Applied Sciences Leiden and Transmuralis. A multidisciplinary group of professionals and volunteers was involved in the design of the study. Professionals from different organisations participated in the workshops. The personas in the workshops were instrumental to formulating potential solutions for specific situations. For instance, in the case of the man caring for both parents, the persona prompted a discussion regarding the enhancement of respite care and the support provided by the local social team.

Our study contributes to the patient-journey-mapping body of literature in two ways. First, it highlights the multidisciplinary nature of care and support for informal caregivers and people with dementia. Informal caregivers for people with dementia interact with multiple organisations, services and a wide range of professionals. We argue that patient journey mapping should extend beyond healthcare provision alone. Welfare professionals, such as caregiver support workers, may play a key role in supporting informal caregivers and should therefore be included.

Second, when investigating whether and how informal caregivers access and use services, the local context is of particular importance. Describing problems and unmet needs in this context provides an opportunity for identification of context-specific improvements through collaboration with stakeholders, including churches and mosques. In the context of the northern part of the South Holland region, there are no dedicated dementia services or long-term care facilities for people from a migrant background, in contrast to regions that include cities such as Amsterdam, Rotterdam and The Hague. Furthermore, in The Netherlands, the availability of funding and support schemes, for example for home adaptations, may vary between municipalities. For this reason, patient journey mapping is a suitable methodological approach, as it takes into account both the opportunities and constraints of the local context.

Our approach identified opportunities for improvement in both healthcare and welfare provision from the perspectives of informal caregivers and professionals. Joseph et al. (2020) [29] proposed complementing patient journey mapping with dual patient–healthcare provider experience mapping. Although this may be valuable in settings with a stable workforce, its application across multiple organisations and professional groups would substantially increase the complexity and duration of patient journey mapping across the stages of dementia.

A strength of this study is that some informal caregivers provided novel and significant insights into care provision that were previously unknown to most professionals. Almost all participants emphasised the importance of having their voices heard and integrating their experiences into the improvement process.

Another strength is that it resulted in an action plan embraced by professionals. In the process of the patient journey research, the professionals recognised the experiences of the informal caregivers and debated their own experiences. Finally, professionals reached a consensus on investing in six themes: information for clients and professionals; increasing knowledge for current professionals; deployment of key figures; increase in practical culturally sensitive support; a care path; and education for future professionals. By taking into account the opportunities and constraints of the region when identifying solutions, the proposed improvement strategies became realistic and practically applicable. This action plan can improve other developments such as a specific tailored application of telecare to support informal caregivers [30], and cooperation with mental healthcare providers.

In this study, professionals involved in health and social care for people with dementia identified their own limitations and barriers affecting the delivery of care and support. Besides practical problems and a lack of knowledge, they experienced professional uncertainty about how to act in contact with informal caregivers and families as is concordant with other research [7,31]. Ahmad et al. [32] explain this by distinguishing four “framing rules” influencing the relationship between professionals and clients with a migrant background: 1. Proximity within practitioner–client relationships, and the ability to build a relationship of trust; 2. The extent to which the professional is able to respond to the client’s needs in a demand-oriented approach; 3. The fixation on cultural differences and the migrant as “other” and different; 4. The stereotypical assumption that within informal care “they look after their own”. These framing rules are concordant with our findings and underscore the importance of knowledge and education for the professionals to feel more competent.

Informal caregivers had a more comprehensive understanding of the person with dementia because they were familiar with the individual’s life history, significant life events, preferences, and beliefs. In our study, both informal caregivers and professionals indicated a discrepancy between the need for demand-driven care and a supply-oriented approach. To adhere to the national Dementia Guideline [33] and to follow up the recommendations of the former Minister of Health, Welfare and Sport on making the Dutch long-term care for older adults with a migrant background more inclusive [34], it is important to gather prevalence data regarding dementia among this population. When prevalence data is lacking, it may lead to a diminished sense of urgency in addressing the gaps on a practical and strategical level of healthcare reform. Due to demographic and epidemiological changes, as well as personnel shortages in care provision, professionals may experience a discrepancy between following the guideline and their own capabilities to work accordingly. Intersectoral and interprofessional collaboration may relieve this tension. Applied sciences can facilitate discovering gaps, understanding different perspectives and problem-solving in the field [35].

This study has several limitations. We were unable to include individuals with dementia and a migrant background themselves and hear their experiences and wishes. Although we did not use the word dementia in the conversation, informal caregivers might have been hesitant or afraid that talking about feelings and experiences was challenging for their family member, triggering different emotions [31]. Another explanation could be the lack of the same cultural background and language of the researcher. First-generation older migrants might prefer interviewers of the same ethnic origin or who speak their native language [36]. Some informal caregivers stated that they were themselves hesitant to confide in someone of their own cultural background because of the possibility of gossip and negative remarks. Other reasons for not consenting interviewing older persons with dementia could be conflicting perspectives within the family on this topic.

Another limitation is that the informal caregivers were not involved in the design of the study. We also had a limited number of participants. Previous research has consistently shown that recruiting older migrants and their informal caregivers is challenging [36,37]. Many informal caregivers for people with a migrant background and dementia remain outside the view of professional care providers [24]. In our study, professionals, such as case managers, made considerable efforts to recruit these informal caregivers. However, most informal caregivers experienced difficulties balancing caregiving responsibilities with paid employment and caring for their own families. As a result, they often had insufficient time or energy to participate in an interview.

Furthermore, we do not have a representative selection of the different cultural backgrounds in the region. We were unable to include informal caregivers with a Turkish background, despite the substantial presence of older people with this background in the region. This may be important because of the diversity of older people with dementia and a migrant background. Successful approaches to include people with a migrant background in research requires a careful approach and an investment in trust with local communities and key figures, looking for a mutually beneficial arrangement for participants and researchers, and awareness from the point of view of the researchers on the methodological challenges and ethical issues [36,37]. We visited the Turkish mosque, a women’s group of Turkish descent, and long before the study started, we discussed the topic of dementia with influential community members from different cultural backgrounds in two cities. Although we tried to build up trust in these relationships in order to include Turkish participants in our study, we were unsuccessful.

We argue that in future research for informal caregivers for people with dementia and a migrant background, generational shifts in knowledge and perceptions on institutional long-term care should be taken into account. However, this may not be the case for all informal caregivers, for example for people with a refugee background. Therefore it is important to distinguish between different groups of informal caregivers for older people with dementia. In our study, some informal caregivers indicated comorbidity of dementia and PTSD due to traumatic events. It is essential that both informal caregivers and healthcare professionals are able to recognise relevant symptoms and respond appropriately, particularly in older adults with dementia who have a refugee background. Future research on this comorbidity should include older people with a migrant background.

A patient journey mapping approach is a valuable method to show positive aspects but also needs for reform in a trajectory of dementia for individuals with a migrant background. The implications for policy and/or practice are to involve the health insurance cooperations and relevant stakeholders from the start of the design of the study. A prerequisite for success is the willingness of all parties involved to follow up recommendations that evolve from the analysis and the embedding of results in policy documents. Moreover, we advise taking sufficient time to set up a guidance group from different expertise and cultural backgrounds, care and welfare professionals as well as from education.

## Figures and Tables

**Table 1:** Sociodemographic characteristics of informal caregivers.

Resp	Age	Gender	Cultural Background	Work	Marital Status	Cares for
R01	40	F	Morocco	No	Married	Father and mother
R02	47	F	Morocco	Yes	Married	Father-in-law
R03	34	F	Curacao	Yes	Married	Father
R04	52	F	Spain	Yes	Married	Mother
R05	30	F	Morocco	Yes	Married	Father
R06	60	F	Morocco	Yes	Married	Mother
R07	57	F	Indonesia	Yes	Single	Mother
R08	59	F	Armenia	Yes	Widow	Father and mother
R09	65	M	Indonesia	No	Single	Mother
R10	62	M	Marocco	PGB ^1^	Married	Father and mother
R11	84	F	Indonesia	No	Married	Partner
R12	58	F	Marocco	No	Married	Father and mother
R13	62	M	Suriname	Yes	Single	Father
R14	33	F	Marocco	Yes	Single	Father

^1^ Fulltime work is informal care with a PGB.

**Table 2:** Needs and bottlenecks from the perspectives of informal caregivers.

Stage	Needs and Bottlenecks
**First signs**	Informal caregivers and families have little knowledge about different forms of dementia. Discussing first signs within families varies because of medical knowledge or stigma. The older generation does not always understand the clinical picture, depending on the country of origin.
2. **Taking action**	Need for respectful assessment of dementia from healthcare providers. Need for a doctor and tests in the language of the person with dementia. Need for translators.
3. **Making contact**	Need for information, for example from the GP. Need for knowledge about the next steps in the illness trajectory. Need for practical information and support for the informal caregiver. Need for comprehensible and timely information about relevant rules, schemes and abbreviations (PGB, WLZ, VPT).
4. **Living at home with dementia**	Need for training about dealing with dementia. Need for training about assistance with daily living activities, such as transfers and lifting techniques. Need for professionals who speak the language of the person with dementia and have the same cultural background. Culturally sensitive healthcare and welfare support. Need for professional care at night. Need for family conversations with professionals about care provisions, instead of conversations with one contact person. Activities in daycare appropriate to the cultural background, for example in the mosque, for men and women. A recognisable atmosphere in daycare that suits different cultural backgrounds. Decisive action by professionals in case of identified health and safety risks. PGB application complexity.
5. **Complex care at home or in a nursing home**	Conversation with family when care becomes too burdensome or terminal care is needed. Need for confirmation and tips for informal care. Need for recognition of informal caregivers’ expertise concerning the person with dementia. Need for housing or a setting for other cultures or religions, especially Islam. Intramural dialogue on perspectives on treatment from an Islamic point of view.
6. **End-of-life care and aftercare for the informal caregivers**	There is no income for the informal caregiver when a PGB stops immediately upon the death of a person with dementia.

**Table 3:** Needs and bottlenecks from the perspectives of professionals.

Stages	Needs and Bottlenecks
**First signs**	It is difficult to become part of a separate and closed community. Establishing contact with a mosque is experienced as difficult.
2. **Taking action**	Language problems. Hiring an interpreter is difficult, funding is unclear. Not everything is interpreted and translated by the children. Feelings of shame within a family and established family role patterns can complicate communication about dementia. Professionals sometimes experience resistance to conveying a diagnosis and families ask for a second opinion. Family members do not always agree with each other about the best approach for care of the person with dementia. Multiple contacts within a family is experienced as problematic for the professional to make appointments. Shifts in the family system make roles complex (caregiver for the same gender of the person with dementia). Awareness of GPs about culturally sensitive tests is unclear.
3. **Making contact**	There is a need for insight into the prevalence of people with dementia and a migrant background in the region. It is unknown if there is an urgent need for facilities or reform. Professionals lack insight into roles and possibilities of different professionals in the region. Case managers would like to have more contact with informal caregivers and the individuals with dementia. Risk management is not always negotiable with informal caregivers. Case managers experience few opportunities to offer culturally appropriate care and sometimes have to apply for a PGB due to the lack of contracts of smaller specific care providers with the regional healthcare office. Professionals experience fragmentation of financial flows. There is a need for short lines of communication between the professionals involved.
4. **Living at home with dementia**	Professionals experience many different opinions within a family about what is considered to be adequate care. Appointments are often rescheduled by informal caregivers. Case managers notice isolation of single caregivers. It is not possible to apply for a PGB for a single caregiver without a family or social network. Daycare for people with dementia will be phased out in nursing homes. It is unclear if municipalities will take over. Admission to a nursing home is poorly negotiable with families due to different aspects. Respite care is currently not sufficient for the informal caregivers.
5. **Complex care at home or in a nursing home**	There is a lack of culturally sensitive care institutions and facilities. There may be a language barrier in nursing homes and at home between professionals and the person with dementia and a migrant background. There is a lack of knowledge and insight into trauma processing (PTSD) in combination with dementia. There is a need for continuing mental healthcare in nursing homes. Caregivers with a PGB receive no income after admission of the person with dementia to a nursing home. There is an extra burden on informal caregivers if two parents need care separately from each other.
6. **End-of-life care and aftercare for the informal caregivers**	Discomfort and a lack of knowledge among professionals concerning rituals around dying and death in other cultures. Families seem to close themselves off from outsiders after the death of the person with dementia. It is unknown if there is a need for guidance and aftercare. Case management stops after the death of the person with dementia. Professionals sometimes experience struggles within a family about choices concerning care. Families are unaware of possibilities for good care within a nursing home. There is no income for the informal caregiver when a PGB stops immediately upon the death of a person with dementia. There is a waiting list for psychological support for caregivers.

## Data Availability

The data supporting the findings of this study are not publicly available due to privacy and confidentiality considerations. Data may be available from the corresponding author upon reasonable request, subject to ethical review and approval by the co-researcher.

## Supplementary Materials

The following supporting information can be downloaded at: https://www.mdpi.com/article/10.3390/geriatrics11040105/s1, Supplementary Material: COREQ checklist [38], Topic list Informal Caregivers.

## References

[1] Parlevliet J.L., Uysal-Bozkir Ö., Goudsmit M., Van Campen J.P., Kok R.M., Ter Riet G., Schmand B., De Rooij S.E. (2016). Prevalence of mild cognitive impairment and dementia in older non-western immigrants in The Netherlands: A cross-sectional study. Int. J. Geriatr. Psychiatry.

[2] (2023). Op weg naar Inclusieve Dementiepreventie. Risicoreductie bij Mensen met een laag Inkomen, Beperkte Gezondheidsvaardigheden en/of een Migratieachtergrond. https://www.pharos.nl/kennisbank/op-weg-naar-inclusieve-dementiepreventie-zorgprofessionals/.

[3] Van Wezel N., Francke A.L., Kayan-Acun E., Devillé W., Van Grondelle N.J., Blom M.M. (2014). Family care for immigrants with dementia: The perspectives of female family carers living in The Netherlands. Dementia.

[4] (2024). Rapport Inclusieve Langdurige Ouderenzorg. https://www.pharos.nl/wp-content/uploads/2024/09/Rapport-Inclusieve-ouderenzorg-2024.pdf.

[5] De Regt S., Fokkema C., Das M. Migrantenouderen in Nederland: Een Beschrijvende Analyse van de Leefsituatie van Ouderen uit de 20 Grootste Herkomstgroepen. Statistische Trends 2022. https://www.cbs.nl/nl-nl/longread/statistische-trends/2022/migrantenouderen-in-nederland.

[6] De Beer J.A., van Duin C., van der Gaag N.L., Ekamper P. Bevolking 2050 in Beeld, Drukker, Diverser en Dubbelgrijs. 2020 NIDI, CBS. https://publ.nidi.nl/output/2020/nidi-cbs-2020-bevolking-2050-in-beeld.pdf.

[7] Nielsen T.R., Nielsen D.S., Waldemar G. (2019). Barriers to post-diagnostic care and support in minority ethnic communities: A survey of Danish primary care dementia coordinators. Dementia.

[8] Selten J., Termorshuizen F., Van Sonsbeek M., Bogers J., Schmand B. (2021). Migration and dementia: A meta-analysis of epidemiological studies in Europe. Psychol. Med..

[9] Gove D., Nielsen T.R., Smits C., Plejert C., Rauf M.A., Parveen S., Jaakson S., Golan-Shemesh D., Lahav D., Kaur R. (2021). The challenges of achieving timely diagnosis and culturally appropriate care of people with dementia from minority ethnic groups in Europe. Int. J. Geriatr. Psychiatry.

[10] Goudsmit M., Van Campen J., Schilt T., Hinnen C., Franzen S., Schmand B. (2018). One Size Does Not Fit All: Comparative Diagnostic Accuracy of the Rowland Universal Dementia Assessment Scale and the Mini Mental State Examination in a Memory Clinic Population with Very Low Education. Dement. Geriatr. Cogn. Dis. Extra.

[11] Stenberg J., Hjelm K. (2023). Migrant informal caregiver perceptions and experiences of caring for a family member with dementia: A systematic review and thematic synthesis. J. Clin. Nurs..

[12] Ahmad M., van den Broeke J., Saharso S., Tonkens E. (2020). Persons with a Migration Background Caring for a Family Member with Dementia: Challenges to Shared Care. Gerontologist.

[13] De Boer A., de Klerk M., Plaisier I. (2021). Mantelzorgers van ouders met een niet-westerse migratieachtergrond. Geron.

[14] De Graaff F.M., Mistiaen P., Devillé W.L., Francke A.L. (2012). Perspectives on care and communication involving incurably ill Turkish and Moroccan patients, relatives and professionals: A systematic literature review. BMC Palliat. Care.

[15] Howard T. (2014). Journey Mapping: A Brief Overview. Commun. Des. Q..

[16] Davies E.L., Bulto L.N., Walsh A., Pollock D., Langton V.M., Laing R.E., Graham A., Arnold-Chamney M., Kelly J. (2023). Reporting and conducting patient journey mapping research in healthcare: A scoping review. J. Adv. Nurs..

[17] Ly S., Runacres F., Poon P. (2021). Journey mapping as a novel approach to healthcare: A qualitative mixed methods study in palliative care. BMC Health Serv. Res..

[18] Mccarthy S., O’raghallaigh P., Woodworth S., Lim Y.L., Kenny L.C., Adam F. (2016). An integrated patient journey mapping tool for embedding quality in healthcare service reform. J. Decis. Syst..

[19] Saragosa M., Nizzer S., Mckay S., Kuluski K. (2023). The hospital-to-home care transition experience of home care clients: An exploratory study using patient journey mapping. BMC Health Serv. Res..

[20] Trebble T.M., Hansi N., Hydes T., Smith M.A., Baker M. (2010). Process mapping the patient journey: An introduction. BMJ.

[21] Barton E., Freeman T., Baum F., Javanparast S., Lawless A. (2019). The feasibility and potential use of case-tracked client journeys in primary health care: A pilot study. BMJ Open.

[22] Kuo S., Huang K.E., Davis S.A., Feldman S.R. (2015). The Rosacea Patient journey: A Novel Approach to Conceptualizing Patient Experiences. Cutis.

[23] Tanner D. (2012). Co-research with older people with dementia: Experience and reflections. J. Ment. Health.

[24] Berdai Chaouni S., De Donder L. (2019). Invisible realities: Caring for older Moroccan migrants with dementia in Belgium. Dementia.

[25] Von Faber M., van der Pas S. (2021). Klantreis dementie. Ervaringen met zorg en ondersteuning van ouderen met dementie en hun mantelzorgers. Geron.

[26] Von Faber M., van der Pas S. (2024). De ‘klantreis dementie’. Het perspectief van professionals voor verbetering van zorg en ondersteuning aan ouderen met dementie en mantelzorgers. Geron.

[27] (2025). Rapport Een ‘Klantreis’ Dementie en een Migratieachtergrond. https://werkplaatssociaaldomeinzhn.nl/siteAssets/downloads/2025/Een%20klantreis%20dementie%20en%20migratieachtergrond.pdf.

[28] Charmaz K. (1990). ‘Discovering’ chronic illness: Using grounded theory. Soc. Sci. Med..

[29] Joseph A.L., Monkman H., Kushniruk A., Quintana Y. (2023). Exploring Patient Journey Mapping and the Learning Health System: Scoping Review. JMIR Hum. Factors.

[30] Mukamal R.C., Gontijo Augusto V., Moraes Dias L., Dias Sarti T., Rego G. (2025). Telemedicine in the Care of Older Adults with Dementia: Caregivers’ Perceptions and Experiences. Geriatrics.

[31] Duran-kiraç G., Uysal-bozkir Ö., Uittenbroek R., Van Hout H., Broese van Groenou M.I. (2023). Informal caregiver and nurse perceptions of access to culturally appropriate health care for ethnic minority persons with dementia: A qualitative study. J. Adv. Nurs..

[32] Ahmad M., Saharso S., Tonkens E. (2025). Framing dementia care in families with a migration background: An analysis of practitioners’ and family caregivers’ views and experiences. Ageing Soc..

[33] Zorgstandaard Dementie (2026). Principeakkoord Versie 23 Januari 2026: Samen Werken aan Ondersteuning en Zorg op maat voor Leven Met Dementie. https://www.zorgstandaarddementie.nl.

[34] Kamerbrief Minister Helder Inclusieve Dementiezorg. https://www.rijksoverheid.nl/documenten/kamerstukken/2024/04/22/kamerbrief-over-aanbieding-rapport-over-inclusieve-ouderenzorg.

[35] Richter S. (2016). Integrated Care for People with Dementia-Results of a Social-Scientific Evaluation of an Established Dementia Care Model. Geriatrics.

[36] Bilecen B., Fokkema T. (2022). Conducting Empirical Research with Older Migrants: Methodological and Ethical Issues. Gerontologist.

[37] Berdai S., De Donder L., Claeys A. (2018). How to involve older people with dementia and their informal caregivers with a migration background in research?. European Congress of Qualitative Inquiry Proceedings.

[38] Tong A., Sainsbury P., Craig J. (2007). Consolidated criteria for reporting qualitative research (COREQ): A 32-item checklist for interviews and focus groups. Int. J. Qual. Health Care.

