# Supplementary material for: Navigating Support for Informal Caregivers of Older Migrants with Dementia in The Netherlands: Insights from Patient Journey Mapping
Source: Geriatrics (Basel). 2026 Aug 14;11(4):105. doi: 10.3390/geriatrics11040105 (PMC13512594; doi:10.3390/geriatrics11040105)
Supplement: Supplementary file 1 [file geriatrics-11-00105-s001.zip › Topic list Informal caregivers 010826.pdf]

Topic list - Informal caregivers

1. **Informed consent**

2. **Characteristics of the informal caregiver**
   - Age
   - Other characteristics: marital status, living situation, family composition, employment.
   - What is the health and well-being of the caregiver?
   - Dutch language proficiency and digital skills (for accessing information)
   - Relationship with the person with dementia. Are there more persons to provide care for (both parents)?
   - Cultural background and preferred language of the person with dementia
   - Nature and hours of caregiving
   - Other caregivers and their roles in caregiving

3. **Description of the journey**

Current phase- *Could you describe the current situation and what daily life looks like for you?*

- Current stage of the illness trajectory
- Informal care / professional care
- Sources of support
- Emotions
- Positive experiences? Are there challenges, needs, wishes?

From this point onward, discuss earlier phases and future expectations

1st signs - *Can you tell me about the first time you noticed that something had changed?*

- What changes were noticed (memory, behavior, speech)? (unusual behavior, dangerous behavior, out of character)?
- Meaning of that change, by caregiver, person with dementia and others (family, network):
  - knowledge/understanding of dementia: influence of culture/religion/language: (cultural explanation e.g. 'confusion, crazy, forgetful, old' 'mental illness' / biomedical e.g. Alzheimer's / no word for dementia in the person's native language)
  - Emotions
- How communicated to the person with dementia; which words were used?
- Expectations regarding providing informal care? (within the family, community or culture?)
- Positive experiences ? Problems? What did you need? From whom? [emotions]

What action has been undertaken? *Can you tell me what happened after realizing something was wrong?*

- Has the GP been consulted?
- Has a diagnosis been established?
- If yes: Why? When? By whom? How was a diagnosis established? In which language was the assessment? What was told to the person with dementia and by whom?
- If not: what were the reasons?
- Emotions
- Positive experiences? Problems? What did you need? From whom?

Providing care at home - *Can you tell me about how care is/has been provided at home?*

- Who provides which aspects of care (coordinating tasks, executive care tasks, assistance, legal/financial matters)?
- Are other persons involved (church, mosque)?

- Use of a Personal Budget (PGB)?
- Emotions
- Positive experiences? Problems? What is needed? From whom?

<u>Contact with professional care</u> - *Could you tell me about your experiences with professional care?*

- Which professionals were/are involved during the journey?
- Use of home care / adult day care services: is this an option (why or why not)? What elements are important in care and support?
- Who is the main contact person for professionals?
- Use of assistive technology (domotica)?
- Positive experiences? Problems? What is needed? From whom?

<u>In the event of further deterioration and complex care</u>- *Can the informal care be sustained now and in the future?*

- Is admission to a long-term care facility of nursing home considered an option? Considerations?
- Are there personal limits to informal caregiving?
- Emotions
- Positive experiences? What is needed? From whom?

<u>Future</u> - *As the illness progresses, how do you think about the future?*

- Expectations regarding the further progression of the illness and the process of care (hopes, worries, wishes, needs).

<u>If the person with dementia has died</u>- *Looking back, could you tell me about the final phase of ....life?*

- How was care provided? Was aftercare provided for the informal caregiver(s)?
- Emotions
- What were the experiences of the informal caregiver(s)? Positive experiences, negative experiences and unmet needs?

<u>Finally, in conclusion</u>- *Do you have recommendations for improvement?*

- Recommendations for professional care and support for people with a migration background and dementia.
- Recommendations for professionals regarding support for informal caregivers.
- Advice for other informal caregivers with a similar background or in a similar situation.

Is there anything we have not discussed that you think is important for us to know?

Thank the participant for cooperation, and explain the next steps in the timeline of the study
